# Do Patients With Parkinson’s Disease Exhibit Reduced Cheating Behavior? A Neuropsychological Study

**DOI:** 10.3389/fneur.2018.00378

**Published:** 2018-05-24

**Authors:** Nobuhito Abe, Iori Kawasaki, Hiroaki Hosokawa, Toru Baba, Atsushi Takeda

**Affiliations:** ^1^Kokoro Research Center, Kyoto University, Kyoto, Japan; ^2^Sendai Nishitaga National Hospital, Sendai, Japan; ^3^Department of Behavioral Neurology and Cognitive Neuroscience, Tohoku University Graduate School of Medicine, Sendai, Japan

**Keywords:** honesty, morality, Parkinson’s disease, personality, reward

## Abstract

Parkinson’s disease (PD) is a common neurodegenerative disorder characterized by loss of dopamine neurons. Since a seminal report was published in the early twentieth century, a growing body of literature has suggested that patients with PD display characteristic personality traits, such as cautiousness and inflexibility. Notably, PD patients have also been described as “honest,” indicating that they have a remarkable tendency to avoid behaving dishonestly. In this study, we predicted that PD patients show reduced cheating behavior in opportunities for dishonest gain due to dysfunction of the dopaminergic reward system. Thirty-two PD patients without dementia and 20 healthy controls (HC) completed an incentivized prediction task where participants were rewarded based on their self-reported accuracy, affording them the opportunity to behave dishonestly. Compared with HC, PD patients showed significantly lower accuracy in the prediction task. Furthermore, the mean accuracy of PD patients was virtually equivalent to the chance level. These results indicate that PD patients exhibit reduced cheating behavior when confronted with opportunities for dishonest gain.

## Introduction

Parkinson’s disease (PD) is a neurodegenerative disease characterized by clinical symptoms, including bradykinesia, rigidity, resting tremor, and postural instability. PD patients also have impaired cognitive functions, which have a profound impact on quality of life for some of them (1). PD patients exhibit a broad range of cognitive deficits even in the early stages of the disease, and executive dysfunction is the core symptom (2). Studies using standard neuropsychological tests, including the Wisconsin Card Sorting Test (3–9), verbal fluency (10), and Trail Making Test (11, 12), have consistently shown impaired performance in PD patients. Paralleling these frontal lobe deficits, neuroimaging evidence suggests prefrontal hypoperfusion and hypometabolism in PD patients (13, 14).

In addition to motor symptoms and cognitive deficits, certain personality traits have long been noted as characteristic of PD patients. Since the pioneering report by Camp (15), many researchers have used a wide range of personality inventories and questionnaires to identify a specific personality type associated with PD. The general descriptions of PD patients have included nervous, cautious, and rigid (16). A recent meta-analysis reported by Santangelo et al. (17), which included 17 studies evaluating personality traits in PD patients, revealed that the personality profile in PD patients is characterized by high neuroticism and harm avoidance and by low openness, extraversion, and novelty seeking.

Recent neuroimaging studies have shed light on the neural correlates of personality traits in PD patients. For example, Ishii et al. (18) reported that across PD patients, novelty seeking was positively correlated with the connectivity strength of the striatum with the hippocampus and amygdala, and harm avoidance was negatively correlated with the fiber connectivity strength of the striatum, including the ventral area, with the amygdala. Other studies have raised the possibility that regions associated with motor and behavioral control, such as the caudate nucleus and insular cortex, exhibit dysfunction leading to low novelty seeking (19, 20) and disinhibited personality (21). Thus, there is a possible link between personality characteristics and altered dopamine homeostasis, although these studies vary in terms of methodology; moreover, whether the personality traits observed in PD patients are associated with pathological changes in specific brain areas remains under debate (16, 22).

Notably, PD patients have also been described as “honest” (23), indicating that they tend to avoid behaving dishonestly. We previously attempted to clarify the association between ostensible honesty and cerebral dysfunction in PD patients (24). We assumed that PD patients may not choose to avoid telling lies but rather have difficulty lying due to executive dysfunction resulting from pathological changes in prefrontal cortical regions. As expected, compared with healthy controls (HC), PD patients had difficulty providing deceptive responses in a cognitive task. Critically, resting-state ^18^F-fluorodeoxyglucose positron emission tomography (PET) revealed that this difficulty was significantly correlated with hypometabolism in the left dorsolateral and right anterior prefrontal cortices, indicating the critical contribution of these lateral prefrontal regions to deception. These results support the notion that insidious neuropathological changes in PD, especially prefrontal hypometabolism, might underlie ostensible honesty in PD patients.

However, like many previous studies of lying, an important limitation in our previous study was that we instructed participants to lie; therefore, our task involved neither temptation nor morally questionable behavior (25, 26). In recent functional neuroimaging studies, researchers have used a more ecologically valid dishonesty task in which participants are given repeated opportunities to gain money by lying about their accuracy in a prediction task (27–29). In this paradigm, participants are asked to report private information on random self-generated dichotomous outcomes. Reporting one outcome wins participants a reward but reporting the other outcome yields punishment or leaves them empty handed. Therefore, dishonest behavior is indexed by improbably high levels of self-reported accuracy.

Our recent neuroimaging study revealed that people who are sensitive to reward, as characterized by increased blood-oxygenation level-dependent signals in the nucleus accumbens during anticipation of reward, tend to behave dishonestly when confronted with opportunities for dishonest gain (27). Based on this observation, we hypothesized that PD patients in whom the dopaminergic pathway is critically affected exhibit little evidence of cheating due to a disordered reward system. Although some researchers have shown relatively preserved mesocortical dopaminergic transmission in PD patients (30), our hypothesis well fit past studies demonstrating the disruption of both the nigrostriatal and mesocortical dopaminergic pathways (31).

We tested our hypothesis by using a modified, concise version of the dishonesty task to provide PD patients and HC opportunities for dishonest gain. Here, we emphasize that this study has focused on reward processing deficits rather than executive dysfunction. These two kinds of impairments are not necessarily mutually exclusive but reflect different aspects of cognitive deficits. Note that according to previous studies (27–29), we have defined honesty and dishonesty in minimal behavioral terms. Specifically, we have focused on behaviors that are typically regarded as honest or dishonest given the circumstances in the context of monetary rewards. Thus, the results of this study should be interpreted cautiously with awareness of their limited generalizability.

## Methods

The participants were 32 idiopathic PD patients without dementia recruited from the Sendai Nishitaga National Hospital and 20 HC with no history of neurological or psychiatric disease recruited from local communities. The sample size was determined based on our previous neuropsychological study on the ability to provide deceptive responses in PD patients (24). The diagnosis of PD was made by board-certified neurologists according to the UK PD Society Brain Bank criteria (32). The patients’ motor symptoms were evaluated using Hoehn–Yahr staging (33) and the Unified Parkinson’s Disease Rating Scale (UPDRS) part III (34). The inclusion criteria for patients in this study were as follows: aged between 55 and 75 years, age at onset above 40 years, Hoehn–Yahr stage from 1 to 4, and a score of 24 or higher (cutoff for dementia screening) on the Mini-Mental State Examination (MMSE) (35). The exclusion criteria were as follows: a medical history of disease of the central nervous system not directly related to PD (e.g., stroke, head injury, and epilepsy); concurrent psychiatric illness, such as schizophrenia or manic depression; a documented or suspected history of drug abuse and/or alcoholism; a history of deep brain stimulation; anticholinergic medication (trihexyphenidyl); and diabetes mellitus. No patient had dopamine dysregulation syndrome, such as addiction to gambling. PD patients were assessed under usual treatment. The inclusion criteria for HC were as follows: aged between 55 and 75 years and a score of 24 or higher on the MMSE. HC showed no signs of neurological diseases, and the MRI scans detected no gross anatomical abnormalities. The protocol was approved by the Ethical Committee of Sendai Nishitaga National Hospital. All subjects gave written informed consent in accordance with the Declaration of Helsinki.

For all the patients and controls, a set of standard neuropsychological tests was used to identify any explicit cognitive deficits. General cognitive function was assessed by MMSE. Attention was assessed by digit-span subtests from the Wechsler Memory Scale-Revised (36). Frontal lobe function was assessed by the Frontal Assessment Battery (FAB) (37). Table 1 lists the results of the standard neuropsychological tests and statistical comparison between PD patients and HC, as well as the demographic data.

**Table 1:** Demographic and neuropsychological data (mean ± SD) of PD patients and HC.

Variable		PD patients (*n* = 32)	HC (*n* = 20)	*p* Values
**Demographics**				
Age		65.2 (5.3)	66.9 (5.5)	0.27
Sex (female/male)^a^		23/9	8/12	0.02
Education (years)		13.0 (2.3)	12.9 (1.8)	0.76
Duration of PD (years)		5.4 (4.9)	–	–
Levodopa equivalent dose, mg/day		495.0 (316.2)	–	–
Duration of medication (years)		4.1 (4.6)		
UPDRS part III (motor part)^b^		26.3 (15.0)	–	–
Hoehn–Yahr stage (median/range)^c^		2.0/1.0–4.0	–	–
**Cognitive function**				
MMSE (out of 30)		28.2 (2.0)	29.0 (1.5)	0.11
Digit span (WMS-R, out of 12)	Forward Backward	8.2 (1.6) 6.3 (2.2)	8.7 (1.6) 6.7 (1.7)	0.35 0.43
FAB (out of 18)		16.7 (1.2)	17.5 (0.7)	0.005

*A chi-square test was used for the sex ratio, and t-test was used for the remaining variables*.

*^a^No gender difference was found in self-reported accuracy*.

*^b^The UPDRS part III scores were recorded while the patients were “on” medication*.

*^c^n = 1 for stage 1, n = 17 for stage 2, n = 13 for stage 3, and n = 1 for stage 4*.

*UPDRS, Unified Parkinson’s Disease Rating Scale; MMSE, Mini-Mental State Examination; WMS-R, Wechsler Memory Scale-Revised; FAB, Frontal Assessment Battery; PD, Parkinson’s disease; HC, healthy controls*.

**Figure 1:**
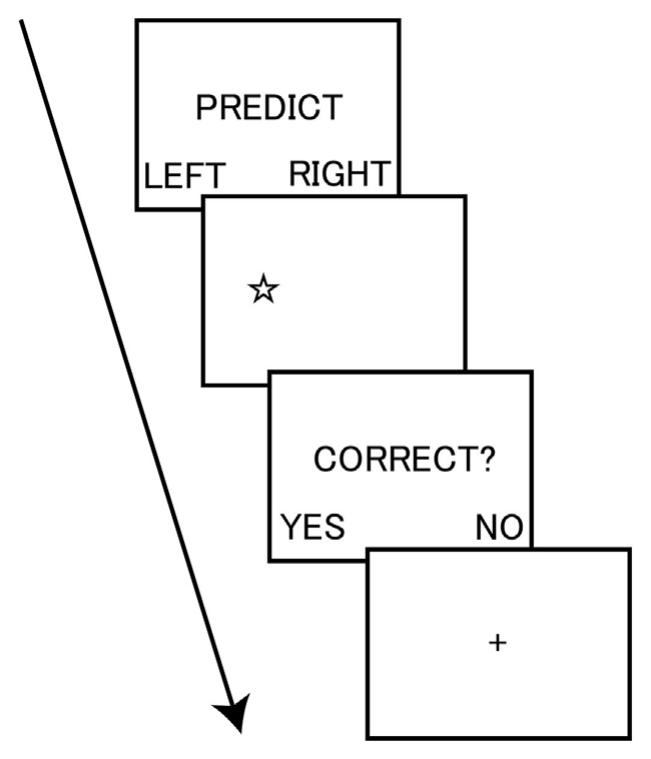
Task sequence of the incentivized prediction task. The participant privately predicted the location of the upcoming stimulus of a star shape. The participant then observed the outcome of the stimulus location (left or right) and indicated whether the prediction was accurate. The task was self-paced.

To measure dishonesty, we used a modified version of the incentivized prediction task. Participants were given an opportunity for dishonest gain by lying about the accuracy of their predictions on whether the stimulus of a star shape was presented on either the left or right side of a computer screen (Figure 1). At the start of each trial, participants were instructed to privately predict the location of the upcoming stimulus. Participants then observed the outcome of the stimulus location (left or right) and were asked to indicate whether the prediction was accurate or not. Participants performed the task at their own pace. Critically, before starting the task, participants were informed that they could receive a 500-yen (i.e., approximately 5 USD) coupon toward the purchase of a book if their accuracy, through a total of 20 trials, was higher than the mean accuracy of past participants. Thus, this task afforded participants the opportunity to spontaneously make dishonest moral decisions.

Statistical significance was set at *p* < 0.05 for two-tailed tests. IBM SPSS Statistics version 22 was used for the computations. A chi-square test was used for the sex ratio, and *t*-test was used for the remaining variables. Correlational analyses were performed on the self-reported accuracy and clinical and demographic measures using Pearson’s coefficient.

## Results

**Figure 2:**
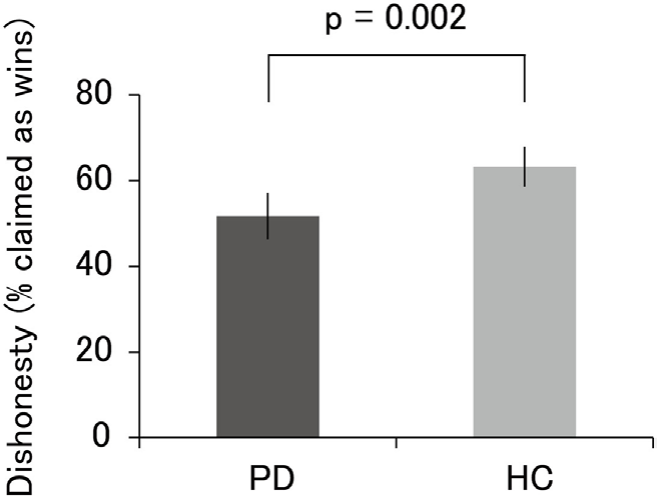
Percentage of self-reported wins in PD patients and HC. The accuracy of PD patients, which did not significantly differ from the chance level of 50%, was lower than that of HC. Error bars represent 95% confidence intervals. Abbreviations: PD, Parkinson’s disease; HC, healthy controls.

The results of the incentivized prediction task are illustrated in Figure 2. The percentage of self-reported wins in PD patients (M = 51.7, SD = 13.6) was significantly lower than that in HC (M = 63.3, SD = 10.5; *t* = 3.23, *p* = 0.002). Even when we used a nonparametric Mann–Whitney *U*-test, the results remained significant (*U* = 166.5, *p* = 0.004). Furthermore, one-sample *t*-test revealed that the percentage of self-reported wins in PD patients did not significantly differ from the chance level of wins (i.e., 50%; *t* = 0.72, *p* = 0.48). The percentage of self-reported wins in HC significantly differed from the chance level of wins (*t* = 5.62, *p* < 0.001). We also used a binomial test to determine whether each participant showed improbably high levels of self-reported accuracy over the chance level of wins. Of the 32 PD patients, only 1 patient was classified as “dishonest” at the individual level, demonstrating that this patient showed significantly higher accuracy than the chance level (*p* < 0.05). Of the 20 HC, 4 participants were classified as dishonest. Thus, at the categorical level, the proportion of participants classified as dishonest was 20% among HC, whereas the proportion of PD patients was only approximately 3%. We emphasize that the low proportion of participants classified as dishonest among HC in this study is not inconsistent with the proportion reported in previous neuroimaging studies (i.e., ~40%) (27–29). Taken together, these results indicate that HC behaved dishonestly, at least to some extent, whereas PD patients exhibited little evidence of cheating.

We also assessed whether the percentage of self-reported wins in PD patients was correlated with other clinical and demographic measures. No significant correlation was observed among these independent variables and dishonesty (age, *r* = 0.01, *p* = 0.94; education, *r* = 0.03, *p* = 0.87; duration of PD, *r* = 0.20, *p* = 0.28; levodopa equivalent dose, *r* = 0.08, *p* = 0.66; duration of medication, *r* = 0.24, *p* = 0.19; UPDRS part III, *r* = 0.02, *p* = 0.93; Hoehn–Yahr, *r* = −0.13, *p* = 0.48; MMSE, *r* = 0.24, *p* = 0.19; forward digit span, *r* = 0.20, *p* = 0.26; backward digit span, *r* = 0.29, *p* = 0.10; FAB, *r* = −0.12, *p* = 0.53). We emphasize that the FAB score was not correlated with self-reported accuracy, indicating that the decreased dishonesty level in PD patients measured in this task is not explained by executive dysfunction. Although the sex ratio differed between PD patients and HC in the present sample (χ^2^ = 5.19, *p* = 0.02), there were no sex differences in self-reported accuracy in both PD patients (*t* = 1.65, *p* = 0.11) and HC (*t* = −0.25, *p* = 0.80).

## Discussion

In this study, we used an incentivized prediction task to compare honesty levels between PD patients and HC. The percentage of self-reported accuracy in PD patients was significantly lower than that in HC. Furthermore, the percentage of self-reported wins in PD patients did not significantly differ from the chance level of wins. At the categorical level, the proportion of participants classified as dishonest was 20% in HC, whereas the proportion of PD patients was only approximately 3%. Further analysis revealed that self-reported accuracy was not correlated with frontal lobe function among PD patients. To the best of our knowledge, this study is the first to demonstrate that PD patients show reduced cheating behavior when confronted with opportunities for dishonest gain.

Before discussing the implications of the present findings, one major limitation is worthy of mention. In this study, we did not have a chance to examine medication-dependent performance differences. We speculate that levodopa could have influenced the present effects or even caused the effects entirely. Previous studies have demonstrated an “inverted U” shape relationship between levels of dopamine and dopamine-dependent cognitive abilities (38, 39). Thus, the optimal level of dopamine can increase the frequency of dishonest behavior in PD patients, but both insufficient and excessive levels of dopamine can have adverse effects. Medication-dependent differences in dishonesty should be thoroughly explored using within-subject designs.

With this substantial limitation in mind, our findings have three major implications. First, the present results substantiate our previous functional neuroimaging findings regarding the association between levels of dishonesty and nucleus accumbens activity during reward anticipation. Brain imaging of healthy people cannot provide direct evidence that a certain brain region is necessary for the performance of a specific cognitive task (40). Specifically, nucleus accumbens activation in functional brain imaging studies may reflect brain activity that is not essential for dishonest behavior (27). Therefore, complementary evidence should be obtained from loss-of-function studies. The present neuropsychological findings support the idea that the disordered reward system caused by neuropathological changes in PD is causally relevant to a diminished level of cheating. We believe that the nucleus accumbens is critical for dishonest behavior (27), although we cannot rule out the possibility that other reward-related regions such as the dorsal striatum play a key role in the effects observed in this study.

Second, the present results provide novel insights into ostensible honesty found in PD patients. We propose that PD patients do not behave dishonestly due to their impaired reward anticipation. Consistent with this idea, previous neuropsychological studies have shown that reward-based decision-making, including gambling and effort-based tasks, is affected in PD patients (41–44). In addition, Muhammed et al. (45) recently reported that reward sensitivity, measured by pupillary and saccadic response to monetary incentives, is blunted in PD patients suffering from clinical apathy. Further support for our interpretation comes from a neuroimaging study reported by Pellicano et al. (46); they demonstrated nucleus accumbens volume reduction in PD patients with and without impulse control disorders.

Third, the present results, along with our past neuropsychological study of PD patients regarding deception where prefrontal hypometabolism was associated with difficulty in telling lies (24), provide a more comprehensive picture of ostensible honesty in PD patients. Specifically, our studies indicate that reduced cheating behavior in PD patients is associated with two different types of cognitive impairments, (a) a reward processing deficit (suggested in the present study) and (b) executive dysfunction [reported in our previous study; (24)]. Thus, PD patients are not tempted by dishonest gain due to impaired reward anticipation, and even if they try to cheat, they have difficulty orchestrating deceptive responses due to executive dysfunction. We believe that these two kinds of cognitive deficits are distinct, which is consistent with our finding that self-reported accuracy in the prediction task is not correlated with FAB scores. We speculate that these multiple impairments jointly contribute to formation of classically observed PD-specific honesty.

Another possible explanation for reduced cheating behavior in PD patients is that they do not want to behave dishonestly to avoid the feeling of devaluation that they already have. This idea well fits the recent standard theory of dishonesty put forward by Ariely (47), which assumes that people cheat only a small amount to reap additional rewards while maintaining a positive self-image, although the exact neural mechanisms underlying this effect remain elusive. Testing variables related to apathy and pessimism could have provided us with a broader picture of the behavioral profile of PD patients relevant to dishonest behavior.

Three further limitations of this study warrant attention. First, whether the present findings can be generalized to dishonesty associated with non-monetary rewards remains elusive. Second, our neuropsychological tests are limited, and no direct evidence is available for the link among the patterns of honest and dishonest behavior, cognitive function, and everyday living in PD patients. Assessment of activities of daily living could be informative to better characterize the honesty profile in PD patients. Finally, we did not collect data that can quantify the functional significance of reward-related brain areas, including the nucleus accumbens. Further research should address this issue using an optimized design for obtaining a reliable measure of mesolimbic dopamine neurochemistry, such as PET, to assess dopamine release (48).

## Ethics Statement

The protocol was approved by the Ethical Committee of Sendai Nishitaga National Hospital. All subjects gave written informed consent in accordance with the Declaration of Helsinki.

## Author Contributions

NA developed the study concept and research plan. NA, IK, HH, TB, and AT conducted the research and data analysis. NA wrote the paper. IK, HH, TB, and AT critically revised the paper.

## Conflict of Interest Statement

The authors declare that the research was conducted in the absence of any commercial or financial relationships that could be construed as a potential conflict of interest.

## References

[1] Pillon B Boller F Levy R Dubois B Cognitive deficits and dementia in Parkinson’s disease. 2nd ed In: Boller F Cappa SF, editors. Handbook of Neuropsychology. Amsterdam: Elsevier (2001). p. 311–71.

[2] Abe N Mori E Cognitive impairment in patients with Parkinson disease (Japanese). Brain Nerve (2012) 64:321–31. 22481505

[3] Bondi MW Kaszniak AW Bayles KA Vance KT Contributions of frontal system dysfunction to memory and perceptual abilities in Parkinson’s disease. Neuropsychology (1993) 7:89–102. 10.1037/0894-4105.7.1.89

[4] Bowen FP Kamienny RS Burns MM Yahr M. Parkinsonism: effects of levodopa treatment on concept formation. Neurology (1975) 25:701–4. 10.1212/WNL.25.8.701 1171402

[5] Brown RG Marsden CD. Internal versus external cues and the control of attention in Parkinson’s disease. Brain (1988) 111:323–45. 10.1093/brain/111.2.323 3378139

[6] Brown RG Marsden CD An investigation of the phenomenon of “set” in Parkinson’s disease. Mov Disord (1988) 3:152–61. 10.1002/mds.870030207 3221902

[7] Gotham AM Brown RG Marsden CD. ‘Frontal’ cognitive function in patients with Parkinson’s disease ‘on’ and ‘off’ levodopa. Brain (1988) 111:299–321. 10.1093/brain/111.2.299 3378138

[8] Lees AJ Smith E Cognitive deficits in the early stages of Parkinson’s disease. Brain (1983) 106:257–70. 10.1093/brain/106.2.257 6850270

[9] Taylor AE Saint-Cyr JA Lang AE Frontal lobe dysfunction in Parkinson’s disease. The cortical focus of neostriatal outflow. Brain (1986) 109:845–83. 3779372 10.1093/brain/109.5.845

[10] Henry JD Crawford JR. Verbal fluency deficits in Parkinson’s disease: a meta-analysis. J Int Neuropsychol Soc (2004) 10:608–22. 10.1017/S1355617704104141 15327739

[11] Pirozzolo FJ Hansch EC Mortimer JA Webster DD Kuskowski MA. Dementia in Parkinson disease: a neuropsychological analysis. Brain Cogn (1982) 1:71–83. 10.1016/0278-2626(82)90007-0 6927555

[12] Reitan RM Boll TJ Intellectual and cognitive functions in Parkinson’s disease. J Consult Clin Psychol (1971) 37:364–9. 10.1037/h0032005 5121817

[13] Hosokai Y Nishio Y Hirayama K Takeda A Ishioka T Sawada Y Distinct patterns of regional cerebral glucose metabolism in Parkinson’s disease with and without mild cognitive impairment. Mov Disord (2009) 24:854–62. 10.1002/mds.22444 19199357

[14] Kikuchi A Takeda A Kimpara T Nakagawa M Kawashima R Sugiura M Hypoperfusion in the supplementary motor area, dorsolateral prefrontal cortex and insular cortex in Parkinson’s disease. J Neurol Sci (2001) 193:29–36. 10.1016/S0022-510X(01)00641-4 11718747

[15] Camp CD Paralysis agitans, multiple sclerosis and their treatment. In: White WA Jelliffe SE Kimpton H, editors. Modern Treatment of Nervous and Mental Disease. Philadelphia: Lea & Febiger (1913). p. 651–7.

[16] Ishihara L Brayne C. What is the evidence for a premorbid parkinsonian personality: a systematic review. Mov Disord (2006) 21:1066–72. 10.1002/mds.20980 16755553

[17] Santangelo G Garramone F Baiano C D’Iorio A Piscopo F Raimo S Personality and Parkinson’s disease: a meta-analysis. Parkinsonism Relat Disord (2018) 49:67–74. 10.1016/j.parkreldis.2018.01.013 29358028

[18] Ishii T Sawamoto N Tabu H Kawashima H Okada T Togashi K Altered striatal circuits underlie characteristic personality traits in Parkinson’s disease. J Neurol (2016) 263:1828–39. 10.1007/s00415-016-8206-0 27334907

[19] Kaasinen V Aalto S Nagren K Rinne JO. Insular dopamine D2 receptors and novelty seeking personality in Parkinson’s disease. Mov Disord (2004) 19:1348–51. 10.1002/mds.20191 15389994

[20] Menza MA Mark MH Burn DJ Brooks DJ Personality correlates of [18F]dopa striatal uptake: results of positron-emission tomography in Parkinson’s disease. J Neuropsychiatry Clin Neurosci (1995) 7:176–9. 10.1176/jnp.7.2.176 7626960

[21] Lawrence AD Brooks DJ Whone AL. Ventral striatal dopamine synthesis capacity predicts financial extravagance in Parkinson’s disease. Frontiers Psychol (2013) 4:90. 10.3389/fpsyg.2013.00090 23450713 PMC3583186

[22] Santangelo G Piscopo F Barone P Vitale C. Personality in Parkinson’s disease: clinical, behavioural and cognitive correlates. J Neurol Sci (2017) 374:17–25. 10.1016/j.jns.2017.01.013 28087060

[23] Menza M. The personality associated with Parkinson’s disease. Curr Psychiatry Rep (2000) 2:421–6. 10.1007/s11920-000-0027-1 11122991

[24] Abe N Fujii T Hirayama K Takeda A Hosokai Y Ishioka T Do parkinsonian patients have trouble telling lies? The neurobiological basis of deceptive behaviour. Brain (2009) 132:1386–95. 10.1093/brain/awp052 19339257 PMC2677797

[25] Abe N. The neurobiology of deception: evidence from neuroimaging and loss-of-function studies. Curr Opin Neurol (2009) 22:594–600. 10.1097/WCO.0b013e328332c3cf 19786872

[26] Abe N. How the brain shapes deception: an integrated review of the literature. Neuroscientist (2011) 17:560–74. 10.1177/1073858410393359 21454323

[27] Abe N Greene JD. Response to anticipated reward in the nucleus accumbens predicts behavior in an independent test of honesty. J Neurosci (2014) 34:10564–72. 10.1523/JNEUROSCI.0217-14.2014 25100590 PMC6802594

[28] Greene JD Paxton JM. Patterns of neural activity associated with honest and dishonest moral decisions. Proc Natl Acad Sci U S A (2009) 106:12506–11. 10.1073/pnas.0900152106 19622733 PMC2718383

[29] Yoneda M Ueda R Ashida H Abe N. Automatic honesty forgoing reward acquisition and punishment avoidance: a functional MRI investigation. Neuroreport (2017) 28:879–83. 10.1097/WNR.0000000000000848 28746066 PMC5585130

[30] Sawamoto N Piccini P Hotton G Pavese N Thielemans K Brooks DJ. Cognitive deficits and striato-frontal dopamine release in Parkinson’s disease. Brain (2008) 131:1294–302. 10.1093/brain/awn054 18362097

[31] Monchi O Petrides M Mejia-Constain B Strafella AP. Cortical activity in Parkinson’s disease during executive processing depends on striatal involvement. Brain (2007) 130:233–44. 10.1093/brain/awl326 17121746 PMC3714298

[32] Gibb WR Lees AJ. The relevance of the Lewy body to the pathogenesis of idiopathic Parkinson’s disease. J Neurol Neurosurg Psychiatry (1988) 51:745–52. 10.1136/jnnp.51.6.745 2841426 PMC1033142

[33] Hoehn MM Yahr MD Parkinsonism: onset, progression and mortality. Neurology (1967) 17:427–42. 10.1212/WNL.17.5.427 6067254

[34] Fahn S Elton R Unified Parkinson’s Disease Rating Scale (Vol. 2). Florham Park, NJ: Macmillan Healthcare Information (1987).

[35] Folstein MF Folstein SE McHugh PR “Mini-mental state”. A practical method for grading the cognitive state of patients for the clinician. J Psychiatr Res (1975) 12:189–98. 10.1016/0022-3956(75)90026-6 1202204

[36] Wechsler D Wechsler Memory Scale-Revised. San Antonio: Psychological Corporation (1987).

[37] Dubois B Slachevsky A Litvan I Pillon B. The FAB: a Frontal Assessment Battery at bedside. Neurology (2000) 55:1621–6. 10.1212/WNL.55.11.1621 11113214

[38] Cools R Barker RA Sahakian BJ Robbins TW. Enhanced or impaired cognitive function in Parkinson’s disease as a function of dopaminergic medication and task demands. Cereb Cortex (2001) 11:1136–43. 10.1093/cercor/11.12.1136 11709484

[39] Mehta MA Swainson R Ogilvie AD Sahakian J Robbins TW. Improved short-term spatial memory but impaired reversal learning following the dopamine D(2) agonist bromocriptine in human volunteers. Psychopharmacology (2001) 159:10–20. 10.1007/s002130100851 11797064

[40] Frackowiak RS Friston KJ Frith CD Dolan RJ Mazziotta J, editors. Human Brain Function. San Diego: Academic Press (1997).

[41] Chong TT Bonnelle V Manohar S Veromann KR Muhammed K Tofaris GK Dopamine enhances willingness to exert effort for reward in Parkinson’s disease. Cortex (2015) 69:40–6. 10.1016/j.cortex.2015.04.003 25967086 PMC4533227

[42] Cools R Barker RA Sahakian BJ Robbins TW. L-dopa medication remediates cognitive inflexibility, but increases impulsivity in patients with Parkinson’s disease. Neuropsychologia (2003) 41:1431–41. 10.1016/S0028-3932(03)00117-9 12849761

[43] Czernecki V Pillon B Houeto JL Pochon JB Levy R Dubois B. Motivation, reward, and Parkinson’s disease: influence of dopatherapy. Neuropsychologia (2002) 40:2257–67. 10.1016/S0028-3932(02)00108-2 12417456

[44] Torta DM Castelli L Zibetti M Lopiano L Geminiani G. On the role of dopamine replacement therapy in decision-making, working memory, and reward in Parkinson’s disease: does the therapy-dose matter? Brain Cogn (2009) 71:84–91. 10.1016/j.bandc.2009.04.003 19442427

[45] Muhammed K Manohar S Ben Yehuda M Chong TT Tofaris G Lennox G Reward sensitivity deficits modulated by dopamine are associated with apathy in Parkinson’s disease. Brain (2016) 139:2706–21. 10.1093/brain/aww188 27452600 PMC5035817

[46] Pellicano C Niccolini F Wu K O’Sullivan SS Lawrence AD Lees AJ Morphometric changes in the reward system of Parkinson’s disease patients with impulse control disorders. J Neurol (2015) 262:2653–61. 10.1007/s00415-015-7892-3 26410743

[47] Ariely D The Honest Truth About Dishonesty: How We Lie to Everyone – Especially Ourselves. New York: HarperCollins Publishers (2012).

[48] Stoessl AJ. Functional imaging studies of non-motoric manifestations of Parkinson’s disease. Parkinsonism Relat Disord (2009) 15(Suppl 3):S13–6. 10.1016/S1353-8020(09)70771-0 20082973

